# Pravastatin suppresses matrix metalloproteinase expression and activity in human articular chondrocytes stimulated by interleukin-1β

**DOI:** 10.1007/s10195-012-0200-4

**Published:** 2012-06-09

**Authors:** Joseph F. Baker, Pauline M. Walsh, Damien P. Byrne, Kevin J. Mulhall

**Affiliations:** 1 School of Medicine and Medical Sciences, University College Dublin, Dublin, Ireland; 2 Orthopaedic Research and Innovation Foundation, Dublin, Ireland; 3 Department of Orthopaedic Surgery, Mater Misericordiae University Hospital, Dublin, Ireland; 4 Department of Orthopaedic Surgery, Cappagh National Orthopaedic Hospital, Finglas, Dublin 21, Ireland

**Keywords:** Articular cartilage, Statins, Matrix metalloproteinases

## Abstract

**Background:**

Matrix metalloproteinases are catabolic enzymes that play a key role in the articular cartilage degeneration evident in degenerative and inflammatory conditions of articular cartilage. The aim of this study is to assess the ability of pravastatin to modify matrix metalloproteinase (MMP) messenger RNA (mRNA) expression and enzyme activity in a culture of normal human chondrocytes stimulated by interleukin-1β.

**Materials and methods:**

Normal human chondrocytes were stimulated with interleukin (IL)-1β for 6 h to induce MMP expression, simulating a catabolic state, and then treated with pravastatin (1, 5 and 10 μM) for a further 18 h before cell lysates and supernatants were harvested. Cells stimulated with IL-1β but not treated with pravastatin served as controls. Real-time polymerase chain reaction (PCR) was used to assess expression of MMP-3 and MMP-9 mRNA. MMP enzyme activity was assessed using a fluorescent MMP-specific substrate. Statistical analysis was performed using analysis of variance (ANOVA).

**Results:**

MMP-3 and MMP-9 mRNA expression was reduced at all concentrations tested with statistically significant trends in reduction (*p* = 0.002 and <0.001, respectively). Analysis of culture supernatants revealed that pravastatin treatment led to a reduction in total MMP activity but not to a statistically significant degree (*p* = 0.07).

**Conclusions:**

Treatment with pravastatin of stimulated human chondrocytes leads to significant down-regulation of selected MMP genes and a non-significant reduction in MMP enzyme activity. Our results provide further evidence that statins may have a role to play in future treatment of disease affecting articular chondrocytes.

## Introduction

3-Hydroxy-3-methylglutaryl-coenzyme A (HMG-CoA) reductase inhibitors, or statins, a class of drug initially developed for treatment of hypercholesterolaemia, appear to offer potential in the treatment of inflammatory diseases including those affecting articular cartilage [1, 2]. Statins have been reported to reduce expression of matrix metalloproteinases (MMPs), catabolic enzymes centrally implicated in the pathophysiology of osteoarthritis [3–5]. MMPs are a zinc-containing, calcium-dependent family of enzymes that can cleave matrix components at physiologic pH and temperature [2, 6]. These enzymes possess all the properties necessary to degrade the cartilage matrix and lead to joint degeneration. MMP-3 in particular has been shown to be a sensitive predictor of joint inflammation and cartilage catabolism in diseased joints [7, 8].

To date, no study has examined the effect of pravastatin, a commonly used statin, on MMP production by human articular chondrocytes. In this study, we aimed to evaluate the effect of pravastatin on MMP expression and MMP activity in a simple in vitro model of articular cartilage catabolism.

## Materials and methods

### Reagents

IL-1β (R&D Systems, USA) was obtained in a lyophilized form and reconstituted with sterile phosphate-buffered saline containing 0.1 % bovine serum albumin. Pravastatin sodium salt (Sigma, UK) was obtained in a lyophilized form and reconstituted with sterile water.

### Cell culture and treatment

Normal human chondrocytes were obtained commercially (Promocell, Germany). The cells were obtained from the normal articular cartilage of a femoral head and cryopreserved. The chondrocytes were cultured in Chondrocyte Growth Media (Promocell, Germany) at 37 °C in a humidified atmosphere of 95 % air and 5 % CO_2_. At passage 4, before cell dedifferentiation, cells were seeded into six-well plates at density of 1 × 10^5^ cells/well and cultured to approximately 80 % confluence prior to treatment.

To investigate the effect of pravastatin treatment, cells were pre-treated with IL-1β for 6 h, and then treated with pravastatin for 18 h at 1, 5 and 10 μM. After a total of 24 h incubation, culture supernatants were removed and stored and cell lysates were collected. Cells cultured in the presence of IL-1β, but without pravastatin, served as experimental controls. All treatments were performed in triplicate.

Stimulation with IL-1β was performed to simulate the pathophysiology of osteoarthritis in which IL-1β is considered a key catabolic stimulant for the production of matrix metalloproteinases [9]. Previous studies focussing on the in vitro response of articular chondrocytes to statins have also used IL-1β as a stimulant of catabolic gene expression and enzyme production, and the treatment times used in this study were also based on these previous similar bodies of work [10–13].

### Cell proliferation analysis

The effect of pravastatin treatment on chondrocyte proliferation was determined using the CellTiter 96^®^ AQueous One Solution Cell Proliferation Assay (Promega, UK). Cells were seeded into 96-well plates at density of 1 × 10^4^ cells/well. At approximately 80 % confluence, cells were treated for 24 h with varying concentrations of pravastatin (1, 5 and 10 μM). Cells cultured in the presence of media, and an equivalent volume of sterile water, served as experimental controls. Following 24 h of treatment, 20 μl phenazine methosulphate solution was added to each well, and this was incubated for 4 h at 37 °C. The absorbance of the culture medium at 490 nm was recorded using a spectrometer. The cell proliferation of treatment cultures was reported as a percentage of control cultures.

### RNA extraction and reverse transcription

Total RNA was extracted from chondrocytes treated with IL-1β or pravastatin using the TRI reagent according to the manufacturer’s instructions (Sigma, Ireland). Contaminating genomic DNA was removed from RNA samples using a DNA-*free*™ kit (Applied Biosystems, UK), and the resulting RNA was then converted to complementary DNA (cDNA) using Enhanced Avian Reverse Transcriptase (Sigma, Ireland).

### Real-time polymerase chain reaction

cDNA served as template for real-time PCR, which was conducted using a QuantiTect SYBR Green PCR kit (Qiagen, UK). Using gene-specific primer pairs, *mmp3* and *mmp9* gene products were measured using absolute quantification and are reported as a function of crossing time (*C*_t_), the cycle number at which PCR amplification becomes linear. mRNA expression was normalised to control and glyceraldehyde-3-phosphate dehydrogenase (GAPDH) expression resulting in mean fold change values. Primers were custom made by Sigma Genosys (nucleotide sequences are presented in Table 1).

**Table 1:** A summary of data for mRNA expression of MMP-3 and MMP-9

	MMP-3 mRNA	MMP-9 mRNA
1 μM	0.75 (0.03)	1.0 (0.01)
5 μM	0.75 (0.02)	0.83 (0.02)
10 μM	0.76 (0.02)	0.72 (0.04)

Values presented are means with standard deviations in parentheses

### Total MMP activity assay

The total MMP activity of culture supernatants was evaluated using the MMP-substrate Mca-Arg-Pro-Lys-Pro-Tyr-Ala-Nva-Trp-Met-Lys(Dnp)-NH_2_ (Bachem, UK) with a quencher and a highly fluorescent end. Activity assays were carried out by incubating 100 μl of substrate (3 μM) with 10 μl of culture supernatant in 0.05 M Tris–HCl, 0.01 M CaCl_2_, 0.15 M NaCl, NaN_3_ (0.02 %), at pH 7.5 for 1 h at 37 °C. The reaction was stopped by addition of 20 μl stopping solution [100 nM ethylenediamine tetraacetic acid (EDTA), NaN_3_ (0.02 %), pH 8.0]. Following cleavage of the quenching moiety, fluorescence was measured at 325 nm excitation and 393 nm emission [14, 15].

### Statistical analysis

All data collected were compiled in an Excel database. Results for mRNA expression and cell viability are demonstrated as a proportion of control (assumed to equal 1). Results from the MMP activity assay are reported as the values recorded. Analysis of variance (ANOVA) testing was used to test for statistically significant trends in the data. Statistical significance was accepted if *p* < 0.05.

## Results

**Fig. 1:**
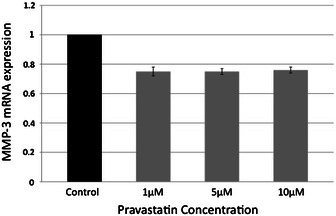
Bar graph illustrating the effect of pravastatin concentration (*x*) on MMP-3 expression (*y*) by normal human chondrocytes after initial stimulation with IL-1β. Gene expression is presented as fold change relative to control. MMP expression was normalised to GAPDH. Error bars represent one standard deviation. *p < 0.05

**Fig. 2:**
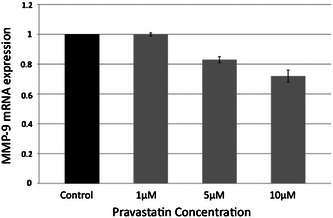
Bar graph illustrating the effect of pravastatin concentration (*x*) on MMP-9 expression (*y*) by normal human chondrocytes after initial stimulation with IL-1β. Gene expression is presented as fold change relative to control. MMP expression was normalised to GAPDH. Error bars represent one standard deviation. *p < 0.05

Pravastatin treatment resulted in a reduction in MMP-3 and MMP-9 mRNA expression. MMP-3 mRNA expression was reduced at all concentrations tested (Fig. 1). ANOVA testing confirmed a statistically significant trend in this data (*p* = 0.002). A statistically significant trend in reduction of MMP-9 expression was also observed (*p* < 0.001) (Fig. 2). Mean values for MMP-3 and MMP-9 mRNA expression are presented in Table 1.

**Fig. 3:**
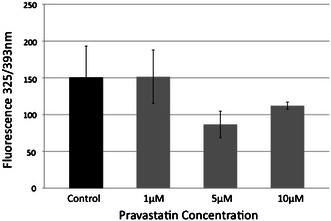
Bar graph illustrating the effect of pravastatin concentration (*x*) on MMP activity (*y*) by normal human chondrocytes after initial stimulation with IL-1β. Cells cultured in the presence of IL-1β only served as an experimental control. The MMP activity of culture supernatants was investigated by cleavage of a fluorescent MMP specific substrate. Data are presented as mean ± standard deviation. *p < 0.05

This reduction in MMP mRNA expression was accompanied by a decrease in MMP activity. Analysis of culture supernatants revealed that pravastatin treatment led to a reduction in total MMP activity (Fig. 3). However, this reduction just failed to achieve statistical significance (*p* = 0.07). Data from the fluorescence assay are presented in Table 2.

**Table 2:** Summary of data from the fluorescence assay determining overall enzyme activity

Control	1 μM	5 μM	10 μM
150.7	151.5	86.7	112.1
42.4	36.3	18.0	4.8

Values presented are means (top) and standard deviations (below)

An analysis of chondrocyte cell proliferation (CellTiter 96^®^ AQueous One Solution Cell Proliferation Assay) revealed that cells treated with pravastatin were found to have a similar rate of proliferation when compared with control cells. Cell viability at 1, 5 and 10 μM concentration was 0.92 (SD 0.03), 0.97 (0.05) and 0.88 (0.02), respectively.

## Discussion

In this study, we investigated the ability of pravastatin to reduce MMP expression and activity by IL-1β-stimulated chondrocytes. The principle finding of this study is that pravastatin treatment led to a reduction in both MMP gene expression and MMP activity, although the MMP activity reduction failed to reach a statistically significant level. The development of agents capable of reducing or inhibiting the expression of MMPs could potentially lead to a slowing down or inhibition of articular cartilage destruction [2, 3].

Statins were originally developed to combat hypercholesterolaemia. However, it has been noted that these drugs also have anti-inflammatory properties, and efforts have been made to apply this potential to the treatment of osteoarthritis (OA). In recent years, a small number of in vitro and animal studies have reported the ability of statins to reduce the gene expression and protein expression of catabolic enzymes implicated in the pathogenesis of osteoarthritis [10, 16]. The treatment of osteoarthritic chondrocytes with simvastatin and mevastatin has shown a reduction in the expression of MMP-3 [10, 12]. Treatment with atorvastatin has been shown to reduce both mRNA and protein expression of MMP-13 [13]. Pravastatin has previously been suggested as a potential treatment based on reduction of selected inflammatory cytokines in a collagen-induced arthritis model in mice [17]. As non-steroidal anti-inflammatory drugs (NSAIDs), the preferred pharmacological agent of patients suffering from osteoarthritis, confer significant gastrointestinal, cardiovascular and renal side-effects, unearthing a more acceptable drug is warranted [18–20].

Pravastatin has been shown to reduce serum levels of C-reactive protein (CRP) [21]. However, it has not yet been tested for its ability to down-regulate MMP gene expression by human chondrocytes nor for its ability to attenuate MMP activity. In this study we found that treatment with pravastatin of IL-1β-stimulated chondrocytes resulted in down-regulation of gene expression of MMP-3 and MMP-9. Each of these MMPs is able to cleave different components of the cartilage matrix. The down-regulation of these MMP genes by pravastatin is consistent with previous research assessing the efficacy of different statins. Lazzerini et al. reported on the ability of simvastatin to attenuate MMP-3 protein expression in cultured osteoarthritic chondrocytes [12]. Simopolou et al. found that treatment with atorvastatin of osteoarthritic chondrocytes resulted in decreases MMP-13 expression at both gene and protein levels [13]. In an animal model of osteoarthritis, Akasaki et al. have shown that intra-articular mevastatin can attenuate histological degradation, and this may be a future therapeutic route of administration [10, 16]. Alternatively they may be used as an oral augment, as has been successfully shown in rheumatoid arthritis, although the pathophysiology of this disease obviously differs [22].

We did notice that a clear dose response was seen in the down-regulation of MMP-9 expression, but we failed to detect a similar dose response with MMP-3. Although we do not have a good explanation for this, it may be related to the massive up-regulation in MMP-3 expression in catabolically stimulated normal articular chondrocytes compared with the significantly lesser up-regulation of other matrix metalloproteinases [23]. We also note that the reduction in mRNA production did not necessarily result in a corresponding decrease in MMP activity. As we used a non-specific measure of MMP activity, it is plausible that other enzymes from the MMP class are not suppressed and remain active. Further work can clarify which other MMP members are inhibited by pravastatin.

We acknowledge that this is an in vitro assessment and that in vitro results can be readily criticised for not accurately replicating the in vivo process. However, monolayer culture remains a reasonable starting point for assessment of new treatment options and the best possible method available. We must also acknowledge that the pathophysiology of various articular cartilage diseases differs and IL-1β is not the only driver of catabolic activity. The use of other stimulants [such as tumour necrosis factor (TNF)-α for example] may be of assistance, but this would also add an extra layer of complexity to the analysis when study of statins as a potential treatment is still just being explored.

In summary, using an in vitro model, we have found that treatment with pravastatin results in a reduction of MMP-3 and MMP-9 mRNA expression and MMP enzyme activity. These findings provide further support for use of statins as a potential treatment of diseases of articular cartilage in which there is an inflammatory or catabolic component to the disease process. Although further work is clearly warranted, this may represent an exciting juncture in the development of new treatments.
